# Correlation of ER, PR, and HER2 at the protein and mRNA levels in Asian patients with operable breast cancer

**DOI:** 10.1042/BSR20211706

**Published:** 2022-01-18

**Authors:** Chih-Jung Chen, Ting-Hao Chen, Jason Lei, Ji-An Liang, Po-Sheng Yang, Chiun-Sheng Huang, Chia-Ming Hsieh, Ling-Ming Tseng, Liang-Chih Liu, Skye Hung-Chen Cheng, Kuan-Hui Shih

**Affiliations:** 1 Department of General Surgery, China Medical University Hospital, Taichung, Taiwan; 2 Department of Medical Operation, Amwise Diagnostics Pte. Ltd., Singapore; 3 Institute of Epidemiology and Preventive Medicine, National Taiwan University, Taipei, Taiwan; 4 Department of Product Development, Amwise Diagnostics Pte. Ltd., Singapore; 5 Department of Radiation Oncology, China Medical University Hospital, Taichung, Taiwan; 6 Department of General Surgery, MacKay Memorial Hospital, Taipei, Taiwan; 7 Department of Surgery, National Taiwan University Hospital, Taipei, Taiwan; 8 Department of General Surgery, Taiwan Adventist Hospital, Taipei, Taiwan; 9 College of Medicine, National Yang Ming Chiao Tung University, Taipei, Taiwan; 10 College of Medicine, China Medical University, Taichung, Taiwan; 11 Department of Radiation Oncology, Koo Foundation Sun Yet-Sen Cancer Center, Taipei, Taiwan

**Keywords:** Asian population, estrogen, gene expression, HER2, immunohistochemistry, progesterone

## Abstract

Breast cancer is the most common cancer and the leading cause of cancer-related deaths in women. The estrogen receptor (ER), progesterone receptor (PR), and human epidermal growth factor receptor 2 (HER2) are the important biomarkers in the prognosis of breast cancer, and their expression is used to categorize breast cancer into subtypes. We aimed to analyze the concordance among ER, PR, and HER2 expression levels and breast cancer subtyping results obtained by immunohistochemistry (IHC, for protein) and reverse transcriptase-polymerase chain reaction (RT-PCR, for mRNA) and to assess the recurrence-free survival (RFS) of the different subtypes as determined by the two methods. We compared biomarker expression by IHC and RT-PCR in 397 operable breast cancer patients and categorized all patients into luminal, HER2, and triple-negative (TN) subtypes. The concordance of biomarker expression between the two methods was 81.6% (κ = 0.4075) for ER, 87.2% (κ = 0.5647) for PR, and 79.1% (κ = 0.2767) for HER2. The κ-statistic was 0.3624 for the resulting luminal, HER2, and TN subtypes. The probability of 5-year RFS was 0.78 for the luminal subtype versus 0.77 for HER2 and 0.51 for TN, when determined by IHC (*P*=0.007); and 0.80, 0.71, and 0.61, respectively, when determined by the RT-PCR method (*P*=0.008). Based on the current evidence, subtyping by RT-PCR performs similar to conventional IHC with regard to the 5-year prognosis. The PCR method may thus provide a complementary means of subtyping when IHC results are ambiguous.

## Introduction

Breast cancer is the most common cancer and the leading cause of cancer-related deaths in women, with approximately 1.7 million incident cases in 2016 [1]. The estrogen receptor (ER), progesterone receptor (PR), and human epidermal growth factor receptor 2 (HER2) represent critical pathways for tumor growth and replication of breast cancer cells [2]. Molecular subtypes of breast cancer have been established based on the biological expression of these three proteins [3]. They include the luminal (ER/PR-positive and HER2-negative), HER2 (HER2-positive regardless of ER/PR status), and triple-negative (TN; ER-, PR-, and HER2-negative) breast cancer subtypes [3,4].

Surgery to remove the tumor is usually the first line of treatment for breast cancer. To reduce the risk of recurrence, patients can consider adjuvant therapies, including hormonal and HER2-targeted therapies for luminal and HER2-enriched subtypes, respectively [3]. Correct identification of the different subtypes is thus crucial for the management of breast cancer. Conventionally, oncologists identify this important clinical information by estimating ER, PR, and HER2 protein levels using immunohistochemistry (IHC) for staining tumor cells [5,6]. Whereas it is relatively easy to perform IHC, reproducibility issues of the IHC method have been reported, probably due to the subjective nature of both the sampling process and the interpretation and scoring of the staining level [7–9]. Consequently, subtypes could be misidentified, especially in low-volume laboratories [10].

Alternatively, genomic tests have been reported recently in determining the ER (*ESR1*), PR (*PGR*), and HER2 (*ERBB2*) status in breast cancer [10–12]. In contrast with the protein levels determined by IHC, it is the gene expression (mRNA) levels that are determined by the genomic approach, using the reverse transcriptase-polymerase chain reaction (RT-PCR) [13,14]. The reproducibility of the genomic method is very high due to its more homogeneous sampling process, and the automated quantification by instruments without human intervention ensures objectivity [15,16].

In the present study, we compared the ER (*ESR1*), PR (*PGR*), and HER2 (*ERBB2*) status and the resulting subtypes of nearly 400 breast cancer specimens, as determined by the IHC and RT-PCR methods, and then evaluated their corresponding clinical performance in predicting differential breast cancer recurrence.

## Methods

### Study population

**Figure 1:**
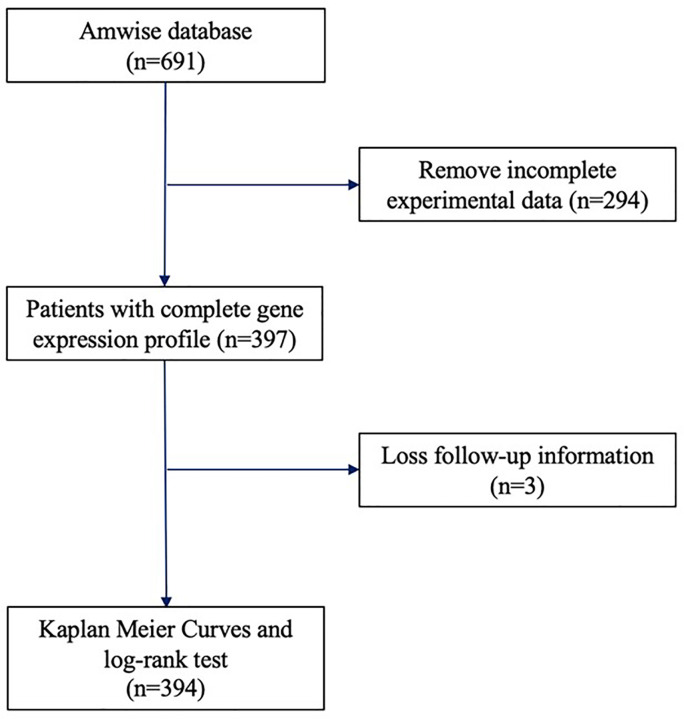
The expression levels of miR-24-3p and IL-1β in AMI patients

The breast cancer patients treated with breast-conserving surgery (BCS) or mastectomy between 2005 and 2016 at multiple medical centers in Taiwan were included in the Amwise dataset (Amwise Diagnostics Pte. Ltd.). Figure 1 shows the subject selection process. The institutional review board of each participating medical center approved the study protocol. The inclusion criteria were (1) with invasive breast cancer; (2) had received mastectomy or BCS as first treatment; (3) with ER, PR, and HER2 status confirmed by IHC and/or fluorescence *in situ* hybridization (FISH) at each participating center; and (4) with formalin-fixed paraffin embedded (FFPE) tissue sections for RT-PCR testing. Patients at a stage of N3 or M1 were excluded.

### Determination of the ER (*ESR1*), PR (*PGR*), and HER2 (*ERBB2*) status

The ER, PR, and HER2 results from the IHC/FISH testing were obtained from the medical charts of each participating hospital and were considered the gold standard for positive/negative expression and subtype determination. For the determination of positive/negative expression of the *ESR1*, *PGR*, and *ERBB2* genes, receiver operating characteristic (ROC) curve analysis was used. The cut-off value for positive expression of each of the three genes was determined by the optimal value of both sensitivity and specificity with respect to the IHC results. We normalized the expression of each of the three target genes (i.e., *ESR1*, *PGR*, and *ERBB2*) to the reference genes (*ACTB*, *RPLP0*, and *TFRC*) by a proprietary algorithm, which the cycle threshold (*C*_t_) is the output value from the ABI 7500Fast instrument:

$$\Delta Ct=\frac{25-Ct(ESR1,\;PGR,\;ERBB2)\;+\;[Ct(ACTB)\;+\;Ct(RPLP0)\;+\;Ct(TFRC)]}{3}$$

Breast cancer subtypes were defined as follows: luminal = ER/PR-positive and HER2-negative; HER2 = HER2-positive regardless of ER/PR status; and TN = ER-, PR-, and HER2-negative.

### Reverse transcription polymerase quantitative chain reaction

RNA was extracted from FFPE tissue sections (5–10 µm in thickness) with the RNeasy FFPE Kit (Qiagen, Valencia, CA, U.S.A.). The extracted RNA was stored at −80°C until use after the concentration was determined by OD with a Nanodrop spectrophotometer (Agilent RNA 6000 Nano Kit, Agilent Technologies, Santa Clara, CA, U.S.A.). A total of 2 µg RNA was used for RT-PCR using the RT² First Strand and RT² SYBR Green ROX qPCR MM Kits (Qiagen, Valencia, CA, U.S.A.). Briefly, the RT reaction was performed at 42°C for 15 min before the reaction was terminated at 95°C for 5 min. PCR was performed on the ABI 7500Fast instrument (Thermo Fisher, CA, U.S.A.) using the Standard mode with 40 cycles at 95°C for 15 s and 60°C for 45 s. Primer sequences were as follows: 5′-cacagagaggtcattggttatagag-3′ and 5′-tcacctgtgagagaacagaaac-3′ for *ESR1*; 5′-gagtgggaaagacatttgagagta-3′ and 5′-caggcatacacagatgaaagga-3′ for *PGR*; and 5′-agactgtccctgaaacctagta-3′ and 5′- acaaagcctggatactgacac-3′ for *ERBB2*. For data normalization, primers for three housekeeping genes were also included in the assay: 5′-aatgcttctaggcggactatg-3′ and 5′-ccaatctcatcttgttttctgcg-3′ for *ACTB*; 5′-cttgtctgtggagacggattac-3′ and 5′-ccacaaaggcagatggatca-3′ for *RPLP0*; and 5′-gtacgtgctaacaggctcaata-3′ and 5′- cgagaagacatctcaagaccag-3′ for *TFRC*.

### Concordance and clinical performance of subtyping by the IHC and RT-PCR methods

To evaluate the classification, the concordance between subtype determination using the IHC and RT-PCR methods was analyzed by superimposing the frequency histograms. Concordance rates were analyzed by κ-statistics. κ values of 0.4–0.6 were considered to represent moderate agreement. The clinical performance of the two methods was evaluated by performing Kaplan–Meier survival analysis to measure the probability of recurrence over a 5-year follow-up time for the three different subtypes as determined by the two methods. By using Cox proportional hazards regression for the prognosis of recurrence-free survival (RFS), univariate and multivariate analyses of the two methods were also performed with adjustment for various clinical factors, including age, tumor stage, tumor grade, N stage, and lymphovascular invasion (LVI). All analyses were performed by using R-4.0.2 software, with *P*-values <0.05 considered statistically significant.

## Results

### Characteristics of included patients

A total of 397 operable breast cancer patients were included in the present study (Table 1). Median follow-up was 51.5 months (interquartile range (IQR), 29.5–60.0). Most of the patients (*n*=359, 90.4%) were aged 40 or older. In the subtype classification by IHC, there were 349 (87.9%) luminal patients, 28 (7.1%) HER2-positive patients, and 20 (5.0%) TN. A total of 321 (80.9%) patients exhibited absent or focal LVI, whereas 76 (19.1%) patients showed prominent LVI. Regarding tumor stage, most patients were at T1 (*n*=200, 50.4%) or T2 (*n*=176, 44.3%), with 21 (5.3%) at T3. The majority of patients (*n*=256, 64.5%) were classified as N0, with 125 (31.5%) at stage N1 and 16 (4.03%) at N2. Most patients had grade I (*n*=86, 21.7%) or grade II (*n*=267, 67.3%) tumors, with only 44 (11.1%) at grade III.

**Table 1:** Characteristics of included patients

Characteristics	*n*=397^1^
**Age**	
<40	38 (9.57%)
≥40	359 (90.43%)
**Tumor stage**	
T1	200 (50.38%)
T2	176 (44.33%)
T3	21 (5.29%)
**N stage**	
N0	256 (64.48%)
N1	125 (31.49%)
N2	16 (4.03%)
**LVI**	
No	321 (80.86%)
Yes	76 (19.14%)
**Grade**	
1	86 (21.66%)
2	267 (67.25%)
3	44 (11.08%)
**IHC subtype**	
Luminal	349 (87.91%)
HER2	28 (7.05%)
TNBC	20 (5.04%)
**Relapse**	
No	326 (82.12%)
Yes	71 (17.88%)
Follow-up (months)	51.45 [29.48, 60.00]

Abbreviation: TNBC, triple-negative breast cancer.

^1^Data are presented as *n* (%) or median [25, 75%].

### Cut-off values for gene expression

**Figure 2:**
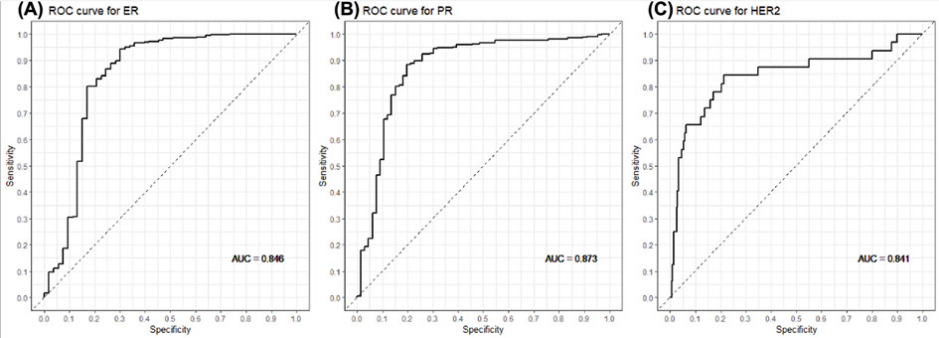
ROC curves for the classification of gene expression by the genomic method (**A**) ROC curve of ESR1 classification of gene expression, (**B**) ROC curve of PgR classification of gene expression, (**C**) ROC curve of ERBB2 classification of gene expression. IHC classification was used as the standard of accuracy.

Cut-off values for the positive expression of the *ESR1*, *PGR*, and *ERBB2* genes were determined to be 22.18667, 18.2038, and 23.69193, respectively. The ROC curves for the determination of ER, PR, and HER2 expression using the genomic method are shown in Figure 2. The area under the curve values for ER (*ESR1*), PR (*PGR*), and HER2 (*ERBB2*) were 0.846, 0.873, and 0.841, respectively.

### Concordance and correlation between the IHC and RT-PCR methods

**Figure 3:**
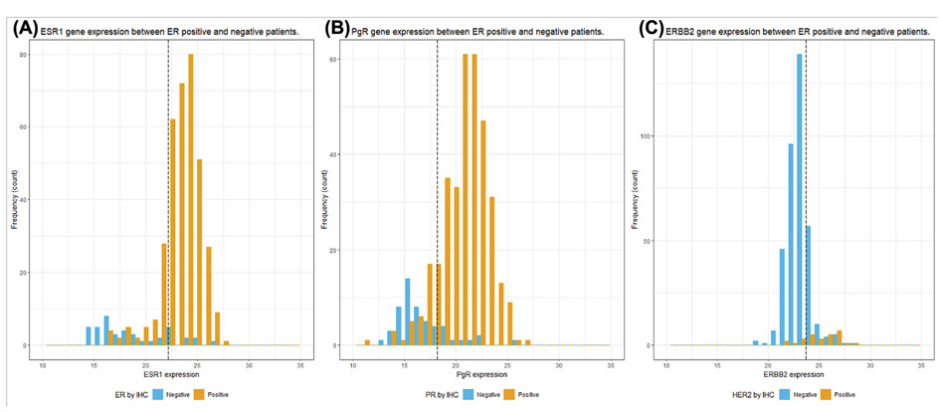
Gene expression of IHC-based positive and negative patients (**A**) The distribution of *ESR1* gene expression between ER-positive and negative patients. (**B**) The distribution of *PgR* gene expression between PR-positive and negative patients. (**C**) The distribution of *ERBB2* gene expression between HER2-positive and negative patients. Dash line: the cut-off value for each gene expression; x-axis: the gene expression after normalization with three housekeeping genes.

In the concordance comparison, concordance between the two methods was analyzed by superimposing the frequency histograms (Figure 3). The corresponding concordance rates were 81.6% (κ = 0.4075, *P*<0.001) for ER (*ESR1*), 87.2% (κ = 0.5647, *P*<0.001) for PR (*PGR*), and 79.1% (κ = 0.2767, *P*<0.001) for HER2 (*ERBB2*) (Table 2). The κ-statistic was 0.3624 (*P*<0.001) for the resulting luminal, HER2, and TN subtypes (Table 3). Altogether, the results indicate that there was moderate agreement between the two methods for the subtyping of breast cancer.

**Table 2:** 2 × 2 table for the concordance between IHC and mRNA expression

	ER by mRNA		Total	κ	*P*-value^1^
	Positive	Negative			
**ER by IHC**				0.4075	<0.001
Positive	288	67	355		
Negative	6	36	42		
**Total**	294	103	397		

	PR by mRNA		Total	κ	*P*-value^1^
	Positive	Negative			
**PR by IHC**				0.5647	<0.001
Positive	301	41	342		
Negative	10	45	55		
**Total**	311	86	397		

	HER2 by mRNA		Total	κ	*P*-value^1^
	Positive	Negative			
**HER2 by IHC/FISH**				0.2767	<0.001
Positive	23	5	28		
Negative	78	291	369		
**Total**	101	296	397		

ER, ^1^from the κ test.

PR, ^1^from the κ test.

HER2, ^1^from the κ test.

**Table 3:** The cross-tabulation of subtype determined by mRNA and IHC

Characteristic	mRNA-based			Total	κ	*P*-value^1^
	Luminal	HER2	TNBC			
**IHC-based**					0.3624	<0.001
Luminal	261	75	13	349		
HER2	4	23	1	28		
TNBC	2	3	15	20		
**Total**	267	101	29	397		

Abbreviation: TNBC, triple-negative breast cancer, ^1^from the κ test.

### Probability of recurrence

**Figure 4:**
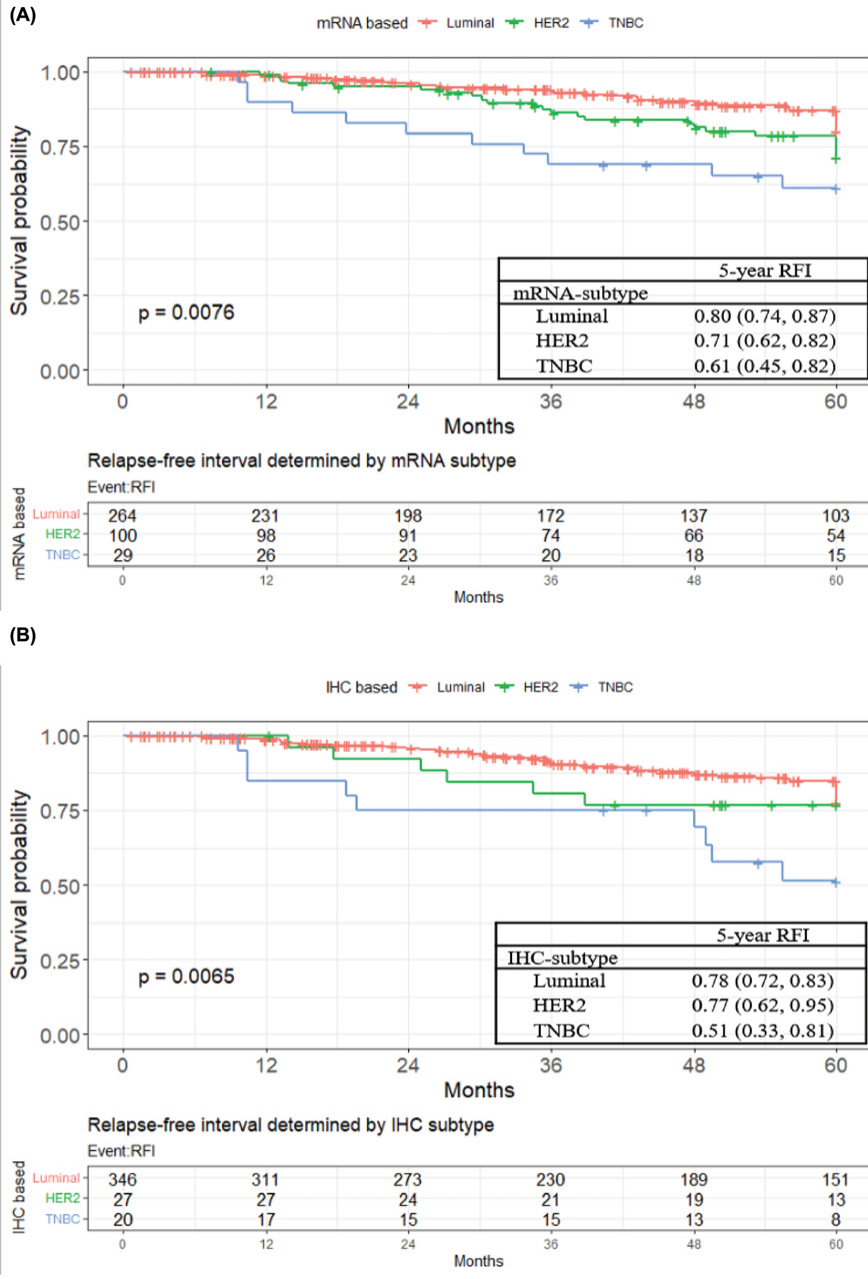
Kaplan–Meier plot for probability of recurrence within 5 years Kaplan–Meier plot for probability of recurrence within 5 years with subtype determined by (**A**) the genomic method or (**B**) the IHC method. (A) The RFS with subtype determined by the genomic method. (B) The RFS with subtype determined by the IHC method.

By Kaplan–Meier survival analysis, the probability of recurrence among the three subtypes was significantly different in both by the RT-PCR (*P*=0.008, Figure 4A) and the IHC (*P*=0.007, Figure 4B) methods. The probability of 5-year RFS in the luminal, HER2, and TN subtypes, as determined by the RT-PCR method, was 0.80 (95% confidence interval (CI): 0.74–0.87), 0.71 (0.62–0.82), and 0.61 (0.45–0.82), respectively (Figure 4A). The probability of 5-year RFS in the luminal, HER2, and TN subtypes, when determined by IHC, was 0.78 (95% CI: 0.72–0.83), 0.77 (0.62–0.95), and 0.51 (0.33–0.81), respectively (Figure 4B).

### Univariate and multivariate Cox proportional hazards’ analyses

Univariate analysis for any recurrence by a Cox proportional hazards model revealed that subtype, either by the IHC or RT-PCR method, was a prognostic factor, especially for the TN subtype (Table 4). By the RT-PCR method, the hazard ratios of the HER2 and TN subtypes were 1.59 (95% CI: 0.94–2.68) and 2.64 (95% CI: 1.33–5.22) when compared with the luminal subtype. In contrast, by the IHC method, the hazard ratios of the HER2 and TN subtypes were 1.27 (95% CI: 0.55–2.97) and 2.89 (95% CI: 1.42–5.85) when compared with the luminal subtype.

**Table 4:** Cox proportional hazards regression model for RFS over 5 years

Characteristics	Univariate			Model 1^1^			Model 2^2^		
	HR	95% CI	*P*-value	HR	95% CI	*P*-value	HR	95% CI	*P*-value
**Age**									
<40	-	-		-	-		-	-	
≥40	0.51	0.27, 0.97	0.039	0.50	0.25, 1.00	0.050	0.46	0.23, 0.93	0.030
**LVI**									
No	-	-		-	-		-	-	
Yes	1.32	0.75, 2.30	0.338	0.76	0.38, 1.53	0.4	0.67	0.33, 1.38	0.3
**Tumor stage**									
T1	-	-		-	-		-	-	
T2	1.41	0.87, 2.29	0.167	1.08	0.63, 1.86	0.8	1.03	0.60, 1.77	>0.9
T3	0.55	0.13, 2.32	0.418	0.30	0.07, 1.35	0.12	0.16	0.03, 0.82	0.028
**N stage**									
N0	-	-		-	-		-	-	
N1	1.23	0.73, 2.08	0.443	1.15	0.63, 2.09	0.6	1.02	0.55, 1.91	>0.9
N2	3.83	1.85, 7.93	<0.001	4.17	1.70, 10.2	0.002	5.14	2.10, 12.6	<0.001
**Tumor grade**									
1	-	-		-	-		-	-	
2	1.74	0.88, 3.46	0.112	1.76	0.84, 3.68	0.13	1.81	0.87, 3.79	0.11
3	3.10	1.36, 7.08	0.007	3.03	1.16, 7.90	0.024	2.93	1.15, 7.45	0.024
**mRNA-based subtype**									
Luminal	-	-		-	-				
HER2	1.59	0.94, 2.68	0.086	1.75	1.02, 3.00	0.043			
TNBC	2.64	1.33, 5.22	0.005	2.21	1.05, 4.62	0.036			
**IHC-based subtype**									
Luminal	-	-					-	-	
HER2	1.27	0.55, 2.97	0.573				1.43	0.60, 3.38	0.4
TNBC	2.89	1.42, 5.85	0.003				4.29	1.85, 9.96	<0.001

Abbreviations: HR, hazard ratio; TNBC, triple-negative breast cancer.

^1^Multivariate Cox proportional hazards regression model including age, LVI, tumor stage, N stage, tumor grade, and mRNA-based subtyping.

^2^Multivariate Cox proportional hazards regression model including age, LVI tumor stage, N stage, tumor grade, and IHC-based subtyping.

In the multivariate analysis, subtyping either by the IHC or the RT-PCR method retained the same trends as in the univariate analysis (Table 4). By the RT-PCR method, the hazard ratios of the HER2 and TNBC subtypes were 1.75 (95% CI: 1.02–3.00) and 2.21 (95% CI: 1.05–4.62), respectively, when compared with the luminal subtype. By the IHC method, the hazard ratios of the HER2 and TNBC subtypes were 1.43 (95% CI: 0.60–3.38) and 4.29 (95% CI: 1.85–9.96), respectively, when compared with the luminal subtype.

## Discussion

Breast cancer is the most prevalent cancer and the leading cause of cancer-related deaths in women [1]. Surgery is usually the first line of treatment, followed by systemic targeted therapy or non-targeted chemotherapy. Targeted therapies include ER-targeted hormonal therapies (tamoxifen, etc.) [17,18] and HER2-targeted monoclonal antibody therapies (herceptin, etc.) [19,20]. The prescription decision is usually based on the ER, PR, and HER2 status. The resulting subtyping information is crucial for the prognosis of the disease.

Subtyping of breast cancer is usually done by IHC staining of tumor cells for ER, PR, and HER2 detection. However, although IHC is relatively easy and inexpensive to perform, subtyping by the IHC method can be error-prone, as selection of the examined areas and assessment of the staining levels could be relatively subjective, leading to misidentification or low reproducibility across different pathologists and laboratories [7,8]. In particular, the IHC staining of HER2 can be ambiguous and requires another experimental approach to validate its results. From the practical perspective, sometimes the determination of HER2 status by using the IHC method is uncertain [21]. For example, if the IHC staining of HER2 is 2+, the FISH method, is required to double-confirm the status of HER2 receptor. Therefore, additional works are necessary to validate the results.

In the current study, we investigated the ER (*ESR1*), PR (*PGR*), and HER2 (*ERBB2*) status by determining their mRNA levels with a genomic (RT-PCR) approach, in contrast to protein-level determination by the IHC approach with antibody staining. The genomic method utilizes the whole tumor tissue for testing, and the expression levels are measured by an automated instrument. Despite the differences in the detection mechanisms, the results of the two methods were in moderate agreement in this cohort with regard to the determination of the ER (*ESR1*), PR (*PGR*), and HER2 (*ERBB2*) levels, and the subsequent subtyping of breast cancer into luminal, HER2- enriched, or TN groups (Figure 2 and Table 2).

It is unclear what caused the discordant results for some cases in the present study, but discordance between the IHC and molecular methods has been reported by others previously [14]. This may be related to the difference in sampling, where the genomic method uses the whole tissue section whereas the IHC method examines only a few portions of the tissue sample. Other possible reasons may include low translational efficiency of mRNA into protein. Nevertheless, based on the Kaplan–Meier analysis of recurrence in the three different subtypes (Figure 3), it is intriguing that both the IHC and RT-PCR approaches performed relatively well in prognosis of RFS of the patients studied. It may thus prove prudent to supplement the IHC with the genomic method for the clinical benefit of patients in selection of appropriate therapies. For example, if the tests are positive with a patient by either the IHC or RT-PCR method, this patient should be considered for a corresponding targeted therapy to avoid undertreatment due to misidentified negative results by the IHC method, or vice versa. In fact, some IHC-negative but RT-PCR-positive patients have been reported to respond to trastuzumab therapy [20].

It should be noted that the present study has limitations. First, the IHC test results were not obtained in a central laboratory setting and thus the criteria may vary in different hospitals. Furthermore, each of the HER2 and TN subtypes represents only a relatively small proportion of the studied population. Lastly, subtypes were generated by approximation, and investigators were not in a setting completely blinded to clinical variables of the studied population.

## Conclusion

Subtyping of breast cancer by complementing IHC tests with genomic tests for determining the ER (*ESR1*), PR (*PGR*), and HER2 (*ERBB2*) status could provide accurate classification results and further treatment of the patients can be based on the subtyping.

## Data Availability

The original contributions presented in the study are included in the article/supplementary material, further inquiries can be directed to the corresponding author.

## Abbreviations

BCS: breast-conserving surgery
CI: confidence interval
ER: estrogen receptor
FFPE: formalin-fixed paraffin embedded
FISH: fluorescence *in situ* hybridization
HER2: human epidermal growth factor receptor 2
IHC: immunohistochemistry
LVI: lymphovascular invasion
PR: progesterone receptor
RFS: recurrence-free survival
ROC: receiver operating characteristic
RT-PCR: reverse transcriptase-polymerase chain reaction
TN: triple-negative
